# A Pediatric Case of Esophageal Foreign Body Removal Facilitated by Structured Briefing and Shared Visualization Using a Video Laryngoscope

**DOI:** 10.7759/cureus.113270

**Published:** 2026-07-24

**Authors:** Sora Seino, Takateru Ihara

**Affiliations:** 1 Department of Pediatrics, Hyogo Prefectural Amagasaki General Medical Center, Amagasaki, JPN

**Keywords:** briefing, coin, educational environment, esophageal foreign body, pediatric, video laryngoscope

## Abstract

Foreign body ingestion is a common pediatric emergency, and coins are among the most frequently ingested objects. When a coin becomes lodged at the pharyngoesophageal junction, prompt removal is required to avoid perforation, mucosal injury, and airway compromise. We report the case of a five-year-old boy with a coin impacted in the upper esophagus at the level of the cricopharyngeus, successfully removed under general anesthesia using a video laryngoscope and Magill forceps without major complications. This case highlights two key educational points. First, the use of a video laryngoscope allowed the operator and supervisor to share the same view in real time, enabling immediate intraoperative guidance. Second, a structured pre-procedural briefing appeared to enhance intraoperative team performance, and a post-procedural debriefing appeared to reinforce learning outcomes. This case suggests that a structured educational approach, combined with shared visualization, may facilitate both safe management and effective teaching of infrequently performed procedures.

## Introduction

Foreign body ingestion is a common pediatric emergency, and coins are among the objects most frequently swallowed by children [1]. The approach to esophageal foreign bodies varies according to the site of impaction. Endoscopic removal is generally recommended for objects lodged distal to the upper esophagus, whereas foreign bodies impacted in the upper esophagus, particularly near the pharyngoesophageal junction or cricopharyngeus, are often removed under direct laryngoscopic visualization with Magill forceps [2]. More recently, a previous report has described removal of upper esophageal foreign bodies using video laryngoscopy [3]. Compared with conventional direct laryngoscopy, video laryngoscopy displays the hypopharynx and esophageal inlet on a monitor, allowing the operator and the supervising physician to share the same view in real time. In emergency settings, procedures with limited opportunities for clinical experience, such as direct removal of esophageal foreign bodies, may benefit from structured team preparation. Previous reports have shown that pre-procedural briefing, in which goals, roles, and risks are explicitly discussed and shared, and post-procedural debriefing, in which the team reviews performance and identifies areas for improvement, can enhance team performance and patient safety [4]. These frameworks are also important for preserving psychological safety and improving learning efficiency [5]. We report a case in which an operator with no prior experience in video laryngoscope-assisted foreign body removal successfully removed an upper esophageal coin on the first attempt. This was facilitated by a structured pre-procedural briefing and real-time supervision through shared video-laryngoscopic visualization.

## Case presentation

**Figure 1:**
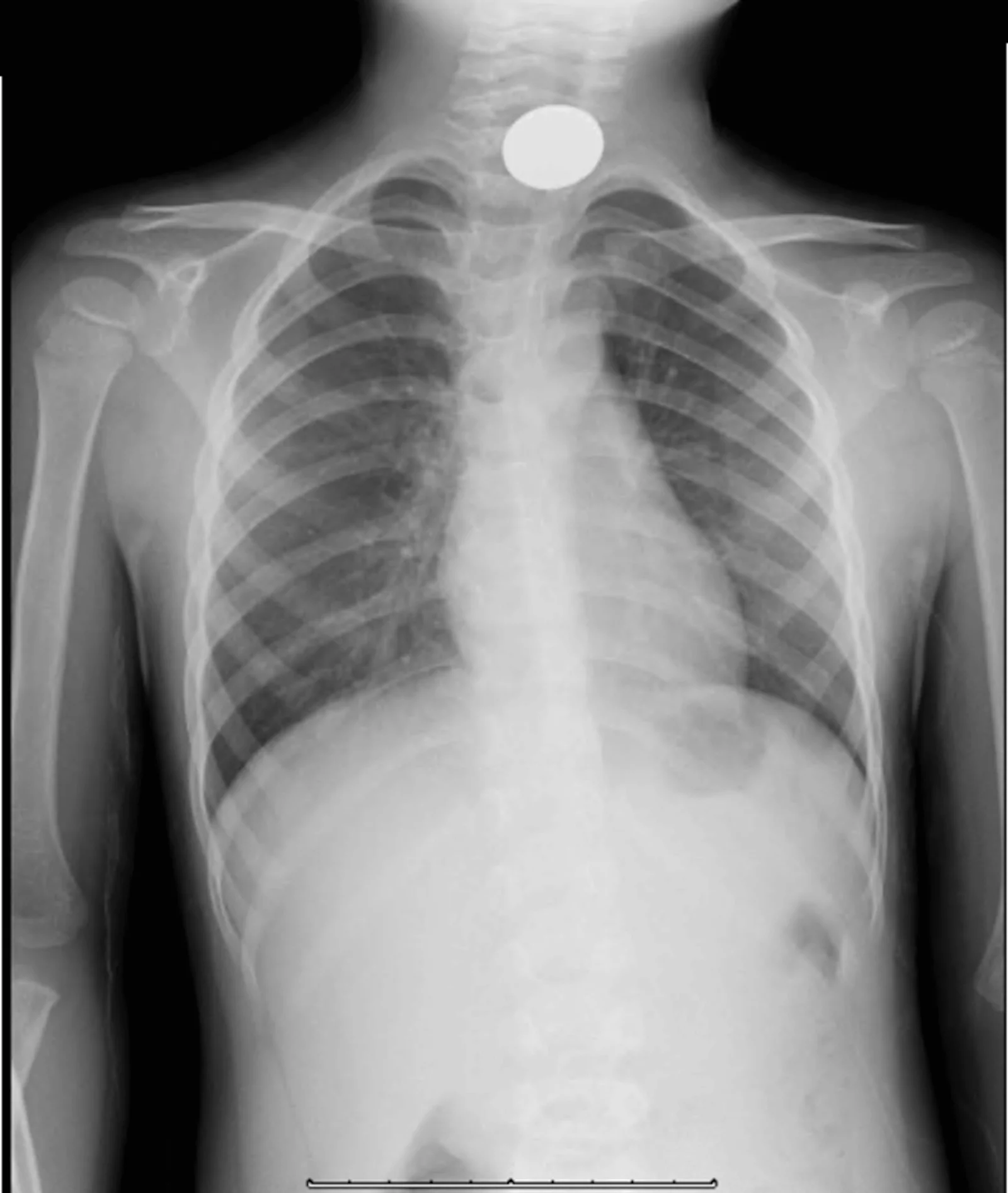
Anteroposterior chest radiograph showing a round coin impacted at the level of the sixth cervical vertebra (C6), corresponding to the cricopharyngeus

A previously healthy 5-year-old boy was brought to the emergency department after his family suspected accidental ingestion of a 10-yen coin (23.5 mm in diameter and 1.5 mm thick) that had been left on a table at home. On arrival, he was alert and had no hypoxemia (peripheral capillary oxygen saturation (SpO2) 98% on room air). His respiratory status was stable, with no increased work of breathing or drooling. His vital signs were as follows: body temperature, 36.8 °C; heart rate, 104 beats/min; and blood pressure, 94/64 mmHg. Physical examination revealed no abnormal findings in the oral cavity and no abnormal breath sounds. Mild pharyngeal erythema and pain were noted; however, no obvious foreign body was seen on visual inspection, and there was no cervical tenderness or swelling on palpation. The abdomen was flat and soft without tenderness, and there were no findings suggestive of gastrointestinal perforation due to foreign body ingestion. Chest radiography demonstrated a round foreign body at approximately the level of the sixth cervical vertebra (Figure 1). The radiograph showed a circular, homogeneous radiopaque object measuring 23.4 mm in diameter without a double-rim sign. Given the clinical history and radiographic findings, the object was consistent with a 10-yen coin.

Because urgent removal was considered necessary, video laryngoscope-assisted foreign body extraction under general anesthesia was planned. Endoscopic removal may require additional preparation, specialized pediatric endoscopic equipment, and the availability of an experienced pediatric endoscopist or a dedicated endoscopy team, which may delay treatment. In contrast, video laryngoscope-assisted removal can be performed promptly without extensive endoscopic equipment by an anesthesiologist or another appropriately trained physician who is skilled in pediatric video laryngoscopy. We therefore selected the video-laryngoscopic approach because suitably trained physicians were immediately available and the procedure could be performed without mobilizing a specialized pediatric endoscopy team. The procedure was performed by a pediatric intensivist skilled in airway management, in the ninth postgraduate year (PGY-9) and with no prior experience in video laryngoscope-assisted foreign body extraction, under the direct supervision of a pediatric emergency physician with extensive experience in this procedure. Anesthesia was managed by another pediatric intensivist experienced in pediatric anesthesia management. A contingency plan was in place to request assistance from a gastroenterologist if removal proved difficult or unsuccessful, and otolaryngology, gastroenterology, and surgery were not directly involved. Although the urgency of the case allowed only limited time, the reported video-laryngoscopic technique [3] and the use of Magill forceps were briefly reviewed with the supervising physician. The procedure was performed at a standard bed in the emergency department. The operator and the supervising physician conducted a structured briefing before the procedure (Table 1).

**Table 1:** Items confirmed during the pre-procedural briefing, organized according to the four categories of the GAPS framework (Goals, Autonomy, Preparation, Strategy), with specific examples from the present case The GAPS framework is used and adapted with permission from its developer, Dr Joel Norton.

GAPS category	Briefing item	Specific example in the present case
Goals	Clarification of the procedural purpose and learning objectives	To remove the coin lodged in the esophagus.
Autonomy	Confirmation of the operator’s experience and level of autonomy	The team confirmed that the operator was experienced in tracheal intubation but had no prior experience in video laryngoscope-assisted foreign body removal, addressed any concerns, and agreed that the supervisor could take over at any time if difficulties arose.
Preparation	Sharing of patient information	The patient’s name, age, body weight, and allergy history were confirmed, and the patient was assessed to have no anticipated difficult airway.
Preparation	Confirmation of equipment and medications to be used	The anesthetic agents to be used, the size of the video laryngoscope, the depth of endotracheal tube fixation, and the equipment to be prepared, including the Magill forceps, as well as their use, were confirmed.
Preparation	Assessment of the risk of complications	The risks of airway and esophageal injury were reviewed.
Strategy	Role assignment	The three physicians were assigned the roles of operator, supervisor, and anesthesia provider.
Strategy	Standardized communication methods	Each physician was encouraged to speak up, and the supervisor paid close attention to how instructions were delivered.
Strategy	Emphasis on safety culture	All team members prioritized the points agreed on during the briefing and carried out the procedure accordingly.

**Figure 2:**
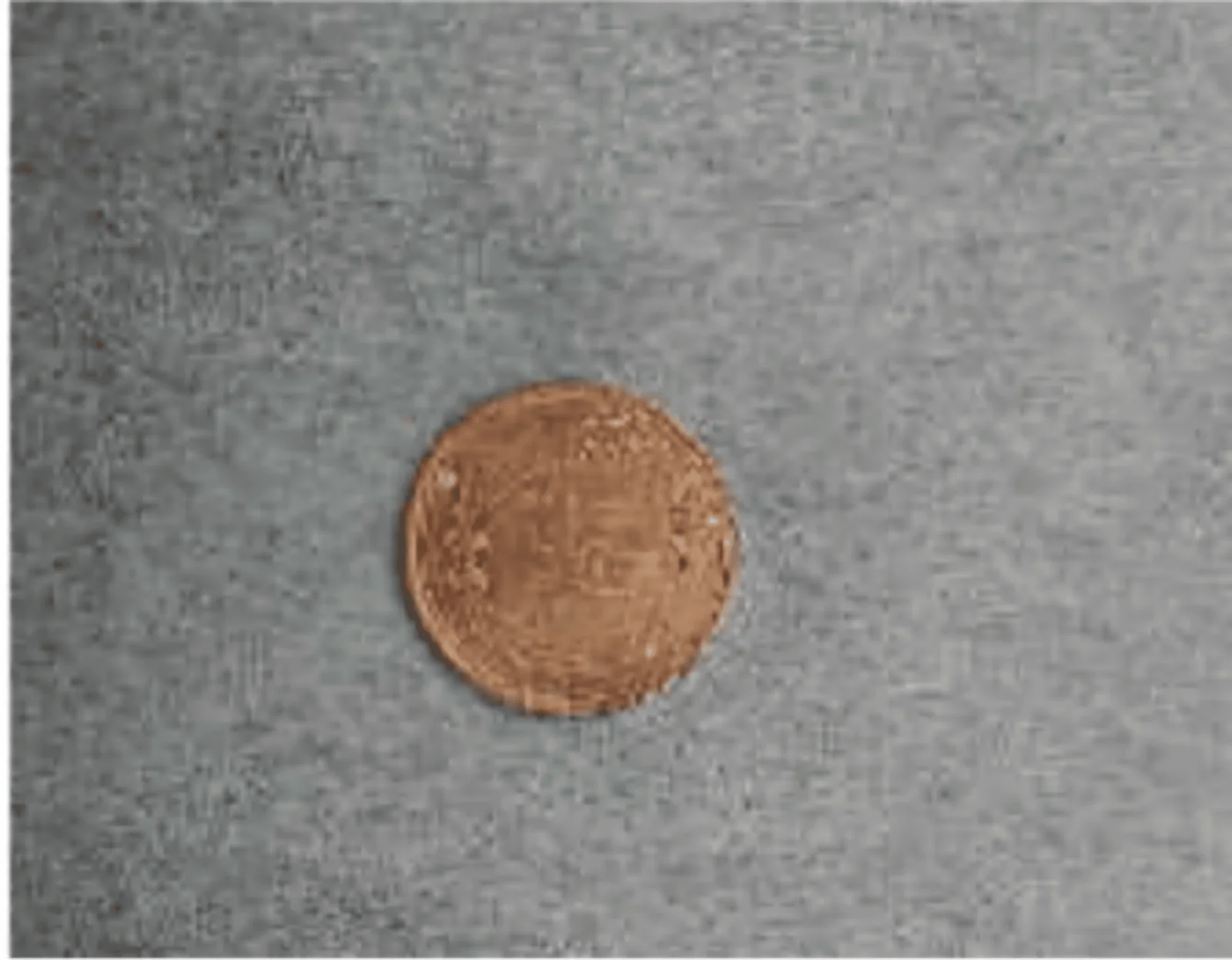
A 10-yen coin removed under video laryngoscope guidance The removal procedure is shown in Video 1.

During this discussion, several points differing from routine tracheal intubation were emphasized: the endotracheal tube should be secured to the left side of the mouth, rather than the right as in routine intubation, to preserve working space for the procedure; the video laryngoscope should be inserted more deeply and with a slightly larger blade than usual; the orientation of the foreign body determines the direction in which it can be grasped and removed; and the forceps should be withdrawn together with the laryngoscope when extracting the object, as the narrow pediatric oral cavity increases the risk of contact between the grasped coin and the laryngoscope blade, which may cause the foreign body to become dislodged during withdrawal. Under general anesthesia with standard endotracheal intubation, the endotracheal tube was secured to the left side of the mouth to preserve working space for the procedure. A video laryngoscope was inserted to elevate the base of the tongue and expose the esophageal inlet. Under shared monitor visualization, Magill forceps were gently advanced into the esophageal opening with the jaws opened vertically to grasp the coin. Because the pediatric oral cavity is narrow and the grasped foreign body can easily come into contact with the laryngoscope blade and become dislodged during withdrawal, the coin was removed together with the laryngoscope as a single unit (Video 1, Figure 2). The extraction required approximately 6 minutes, and the total time from induction of anesthesia to extubation was approximately 15 minutes. No mucosal injury was observed after removal. The patient subsequently awoke without complication, was observed for several hours without complications, and was discharged home the same day after confirming that oral fluid intake was possible. A post-procedural debriefing was then conducted with the team members involved in the case.

**Video 1:** Video laryngoscope-assisted removal of a coin impacted in the esophagus

## Discussion

This case demonstrates that upper esophageal foreign body removal, despite being the operator’s first such procedure, was completed promptly with the aid of a structured pre-procedural briefing and intraoperative supervision through a video laryngoscope. Although the evidence base for briefing has accumulated largely in the context of routine operating-room surgery, the same principles appear applicable to infrequently performed emergency procedures.

Briefing has been reported to improve learner engagement and educational effectiveness by clarifying learning goals and expected performance while preserving psychological safety [6]. In airway management training, briefing has also been shown to improve learner performance and team communication by defining objectives, assigning roles, and reviewing procedural flow in advance [7]. A recent systematic review of 30 studies on preoperative educational briefings extracted recurrent themes from briefing templates and proposed an evidence-based framework consisting of four categories: Goals, Autonomy, Preparation, and Strategy (the GAPS framework), which together encompass trainee goal setting, the degree of learner independence, case-specific preparation, and operative as well as communication strategy [8]. Implementation of briefings structured around these categories has been associated with improvements in surgical learning and performance outcomes, including goal setting, feedback, and trainee autonomy [8]. Guided by this framework, we structured the pre-procedural briefing around the four GAPS categories: clarification of the procedural goal and learning objectives under Goals; confirmation of the operator’s experience and level of autonomy under Autonomy; sharing of patient information under Preparation; and role assignment under Strategy. Because this was an infrequently performed emergency procedure, we additionally incorporated standard perioperative safety-briefing elements (confirmation of equipment and medications, assessment of the risk of complications, standardized communication, and an emphasis on safety culture), consistent with perioperative team briefing, which has been associated with improved team performance and patient safety [4] (Table 1).

Because the GAPS framework was originally derived from briefings for routine scheduled surgery, its application to an infrequently performed emergency procedure, such as upper esophageal foreign body removal, is not fully established. Nevertheless, addressing each of these four categories during the briefing is likely to support both operator learning and procedural performance, with potential value for education and patient safety.

To our knowledge, no prior report has specifically examined the educational impact of intraoperative instruction through video laryngoscopy during esophageal foreign body removal. However, for tracheal intubation, a technically related procedure that requires similar equipment and anatomic orientation, the educational value of video laryngoscopy has been better established [9]. In novice or less experienced operators, video laryngoscopy has been associated with lower failure rates and shorter intubation times, including in simulation settings [9].

In a recent systematic review and meta-analysis, Gunning et al. reported that video laryngoscopy used for teaching was associated with a higher first-pass success rate than conventional direct laryngoscopy and with improved instructional quality and learner confidence because the operator and instructor could view the same anatomy simultaneously [10]. Use of Macintosh-type video laryngoscopes, in particular, has been associated with higher first-pass success and better learner satisfaction and anatomic understanding [11]. These findings support the role of video laryngoscopy as a useful educational tool in airway training.

Direct removal of an esophageal foreign body under laryngoscopic visualization is performed less frequently than tracheal intubation, and no standard simulation method has been established. In this setting, shared visualization through a video laryngoscope, coupled with real-time instruction, may have contributed to first-attempt success in our case. In addition, infrequently performed procedures are known to impose a substantial cognitive load, which may impair recall of steps and precautions [12]. In the present case, important technical points that differed from routine intubation included securing the endotracheal tube to the left side of the mouth to preserve working space, the position at which the blade tip should be applied, and maneuvers to avoid collision between the Magill forceps and the laryngoscope. These details had been emphasized during the pre-procedural briefing, and repeated real-time guidance during the procedure may also have supported successful removal.

This experience should be interpreted with caution. The operator in this case was already experienced in tracheal intubation, familiar with both the video laryngoscope and Magill forceps, and well-acquainted with the relevant anatomy. This likely allowed focused attention on the procedural differences from ordinary intubation such as endotracheal tube depth and extraction mechanics. The extent to which these findings can be generalized to less experienced operators therefore remains uncertain.

For relatively uncommon procedures, such as upper esophageal foreign body removal, retention and consolidation of technical skills are important. Debriefing after the procedure has been shown to enhance educational outcomes by reinforcing technical and non-technical skills, consolidating knowledge, strengthening teamwork, and identifying points for improvement before the next case [13]. Informed by the clinical debriefing literature [14], we conducted a structured post-procedural debriefing covering the following domains, as summarized in Table 2: the conduct of the procedure, technical and non-technical performance, teamwork and communication, complications or safety concerns, improvement measures, action goals for future cases, preservation of psychological safety, and participation by all team members.

**Table 2:** Items to be reviewed during debriefing and specific examples from the present case

Debriefing item	Specific example in the present case
Review of the procedure	The physicians involved in the procedure reviewed the use of the Magill forceps.
Assessment of technical and non-technical skills	The team evaluated how closely the procedure had followed the algorithm established during the pre-procedural briefing.
Analysis of teamwork and communication	The team analyzed whether real-time instruction from the supervising physician had been useful.
Identification of complications and safety concerns	The team discussed the possibility of pushing the coin farther distally and the appropriate timing for aborting the procedure.
Proposal of improvement measures	No specific improvement measures were proposed because the procedure had been completed smoothly.
Setting specific action goals for the next case	The team reviewed the procedural steps and set a goal of using the same instructional approach if a similar case were encountered in the future.
Maintenance of psychological safety	The session began with a brief defusing period, followed by open discussion of participants’ impressions of the procedure; the supervising physician then summarized the strengths and areas for improvement.
Sharing opinions among all participants	The team reviewed the procedural video together and shared their perspectives.

Although simulation and repeated practice remain important for long-term skill retention, appropriate debriefing after an actual clinical case may further strengthen learning [15]. Debriefing has also been associated with improvement in clinical skills, diagnostic reasoning, motivation for learning, and awareness of teamwork [16]. In addition, in operating rooms and emergency care settings, the use of both briefing and debriefing has been associated with better team communication, fewer procedural interruptions or delays, and a stronger culture of patient safety [17]. Our experience supports the importance of an explicit team plan before undertaking such procedures. Ensuring the availability of backup support from otolaryngology, gastroenterology, or surgery is also advisable in case laryngoscopic removal is unsuccessful. Furthermore, no objective educational outcomes, such as skill assessment, procedural time, learner confidence scores, or simulation-based evaluation, were measured in this case; the educational benefits described here therefore remain observational.

## Conclusions

We report the safe and prompt removal of a coin lodged in the upper esophagus of a child using a video laryngoscope and Magill forceps. By providing the operator and the supervising physician with the same clear operative view in real time, the video laryngoscope allowed accurate intraoperative guidance without interrupting the procedure and appeared to support the performance of an operator without prior experience in this technique. This case suggests the potential applicability of a structured educational sequence, consisting of a pre-procedural briefing, shared video-laryngoscopic visualization, and post-procedural debriefing, to an uncommon pediatric emergency procedure such as upper esophageal foreign body removal. Further studies are needed to evaluate the educational and clinical effectiveness of these strategies.
